# Beyond Clinical Factors: Harnessing Artificial Intelligence and Multimodal Cardiac Imaging to Predict Atrial Fibrillation Recurrence Post-Catheter Ablation

**DOI:** 10.3390/jcdd11090291

**Published:** 2024-09-19

**Authors:** Edward T. Truong, Yiheng Lyu, Abdul Rahman Ihdayhid, Nick S. R. Lan, Girish Dwivedi

**Affiliations:** 1 School of Biomedical Sciences, University of Western Australia, Perth, WA 6009, Australia; 23364936@student.uwa.edu.au; 2 Harry Perkins Institute of Medical Research, University of Western Australia, Perth, WA 6009, Australia; yiheng.lyu@research.uwa.edu.au (Y.L.); abdul.ihdayhid@perkins.org.au (A.R.I.); nick.lan@health.wa.gov.au (N.S.R.L.); 3 Department of Computer Science and Software Engineering, School of Physics, Mathematics and Computing, University of Western Australia, Perth, WA 6009, Australia; 4 Department of Cardiology, Fiona Stanley Hospital, Perth, WA 6150, Australia; 5 Curtin Medical School, Curtin University, Perth, WA 6102, Australia; 6 Medical School, University of Western Australia, Perth, WA 6009, Australia

**Keywords:** atrial fibrillation, artificial intelligence, cardiac imaging, catheter ablation, left atrium, machine learning

## Abstract

Atrial fibrillation (AF) is the most common type of cardiac arrhythmia, with catheter ablation being a key alternative to medical treatment for restoring normal sinus rhythm. Despite advances in understanding AF pathogenesis, approximately 35% of patients experience AF recurrence at 12 months after catheter ablation. Therefore, accurate prediction of AF recurrence occurring after catheter ablation is important for patient selection and management. Conventional methods for predicting post-catheter ablation AF recurrence, which involve the use of univariate predictors and scoring systems, have played a supportive role in clinical decision-making. In an ever-changing landscape where technology is becoming ubiquitous within medicine, cardiac imaging and artificial intelligence (AI) could prove pivotal in enhancing AF recurrence predictions by providing data with independent predictive power and identifying key relationships in the data. This review comprehensively explores the existing methods for predicting the recurrence of AF following catheter ablation from different perspectives, including conventional predictors and scoring systems, cardiac imaging-based methods, and AI-based methods developed using a combination of demographic and imaging variables. By summarising state-of-the-art technologies, this review serves as a roadmap for developing future prediction models with enhanced accuracy, generalisability, and explainability, potentially contributing to improved care for patients with AF.

## 1. Introduction

Atrial fibrillation (AF) is a supraventricular tachycardia that arises from uncoordinated electrical signalling within the atria [1]. AF is the most common arrhythmia globally [2], affecting an estimated 46 million individuals worldwide. The incidence and prevalence of AF are projected to increase due to improved screening for AF and an ageing population [1,2,3]. Notably, AF predisposes a patient to an increased risk of mortality and morbidity from cardiovascular disease such as stroke and heart failure [3,4]. Catheter ablation is a well-established rhythm control procedure for patients with AF refractory to antiarrhythmic drugs (AADs). The aim of catheter ablation is to restore and maintain sinus rhythm, leading to subsequent improvements to a patient’s symptoms and quality of life (a summary of guideline indications for catheter ablation in patients with AF is provided in Supplementary Table S1) [5,6,7,8,9]. During the procedure, a catheter is used to deliver radiofrequency, cryogenic, or electrical energy to areas responsible for uncoordinated signalling, isolating local electrical activity. The pulmonary veins (PVs) are the primary targets of catheter ablation for AF because of their known role in initiating the ectopic beats that can cause AF [6].

The efficacy and safety of catheter ablation have been addressed in numerous studies [10,11,12], with catheter ablation demonstrating non-inferiority to use of AADs alone. Moreover, catheter ablation provides greater improvements to patient outcomes, including symptom burden and quality of life, when compared to AADs [13]. While the presence of symptoms and the patient’s functional status can influence the management strategy of patients with AF, studies have yet to demonstrate that catheter ablation reduces risk of death or major adverse cardiovascular events (MACEs) for all patient populations when compared to medical therapy [5,14,15]. However, reductions in all-cause mortality and MACEs have been subsequently demonstrated in subgroup analysis and randomised controlled trials of patients with heart failure [16,17,18]. In light of this, catheter ablation is considered as a worthwhile first-line treatment in specific patient populations, such as patients with symptomatic paroxysmal AF and clinically diagnosed heart failure (Supplementary Table S1) [14]. 

Despite the efficacy of catheter ablation for AF, approximately 35% of patients undergoing catheter ablation for AF will experience AF recurrence within 12 months, and up to 50% of patients will experience recurrence within 5 years [10]. As a semi-invasive procedure with risk of periprocedural complications, AF recurrence is a key endpoint when evaluating the efficacy of catheter ablation. Furthermore, patients who experience AF recurrence after catheter ablation demonstrate an increased burden of AF, as well as increased rates of healthcare utilisation. Hence, there is a growing need to identify patients at increased risk of AF recurrence following catheter ablation [19]. 

AF recurrence in the literature is defined as any recorded atrial tachyarrhythmia (e.g., AF, atrial flutter, atrial tachycardia) lasting more than 30s following a blanking period of typically 3 months after the ablation procedure [20]. Despite this definition, AF recurrence has not been uniformly assessed as an endpoint in research studies, such that there can be considerable variation in how the endpoint of AF recurrence is defined, particularly within retrospective studies [10]. Variations in defining AF recurrence can typically be attributed to differences in the length of blanking periods, monitoring methods used, and monitoring duration. This heterogeneity poses a challenge to comparing recurrence rates between studies. From a mechanistic standpoint, AF recurrence following catheter ablation is also heterogeneous and varied, with distinct differences depending on the time of AF recurrence [21]. The mechanisms for early recurrence of AF, defined as AF recurrence during the blanking period, are unclear and are hypothesised to be the result of periprocedural inflammatory responses [22]. In contrast, long-term AF recurrence is hypothesised to be the result of PV reconnection and the reestablishment of altered electrical signalling [23,24]. However, studies suggest that AF recurrence after 12 months may also have a distinct non-PV-related pathogenic profile [21,25], instead relating to the AF substrate. Despite these hypotheses, many patients experience AF recurrence after catheter ablation without demonstrating inflammation, PV reconnection, or a distinct pathogenic profile, suggesting that our mechanistic understanding of AF recurrence is incomplete [23].

In the absence of a comprehensive understanding of the mechanisms that drive AF recurrence after catheter ablation, it is understood that prediction of AF recurrence is a sensible alternative to optimise and improve patient outcomes. This review will summarise the current state of predicting the recurrence of AF following catheter ablation, providing a discussion spanning from conventional AF recurrence prediction involving univariate predictors and scoring systems to state-of-the-art AF recurrence prediction using the latest advancements in artificial intelligence (AI) and cardiac imaging modalities such as speckle-tracking echocardiography (STE), cardiac computed tomography (CCT), and cardiac magnetic resonance (CMR) imaging. Following this, limitations of these technologies, and future directions for predicting post-catheter ablation AF recurrence will be discussed. 

## 2. Conventional Prediction of AF Recurrence after Catheter Ablation

Given the adverse clinical outcomes associated with AF recurrence following catheter ablation, numerous studies have sought to predict or stratify a patient’s individual risk of AF recurrence [26,27]. Prediction of AF recurrence aids clinicians with both preprocedural and postprocedural decision-making from optimising patient selection to management of patient care post-catheter ablation, especially with regard to the continued use of AADs [8]. It may also aid long-term clinician decision-making regarding the length and frequency of follow up, allowing for improvements in all aspects of patient outcomes, not solely in reducing the rate of AF recurrence.

### 2.1. Predictors of AF Recurrence

For AF recurrence following catheter ablation, predictors can be broadly split into three categories: clinical and demographic variables, biochemical variables, and variables of cardiac anatomy and function. Importantly, these measures are frequently interrelated, both within and between groups [27]. Moreover, AF is a highly comorbid disease, with many patients with AF also suffering from other cardiovascular diseases such as heart failure, or concomitant arrhythmias such as atrial flutter. Hence, identification of these clinical risk factors can be vital in predicting AF recurrence [27]. For example, obstructive sleep apnoea has been identified as an independent predictor of AF recurrence [28]. Epidemiological studies have identified that demographic information such as age and AF type also yield prognostic value with regard to AF recurrence. Specifically, younger patients with paroxysmal AF are most likely to experience long-term freedom from AF after catheter ablation [8,27]. Additional demographic information such as sex, ethnicity, and smoking status have also been identified as potential predictors, but generally to a lesser extent compared to age and AF type [27].

In addition to clinical and demographic variables, research into the implementation of biochemical variables to predict AF recurrence following catheter ablation has increased, with aims to identify novel biomarkers that yield independent predictive value [27]. Examples of biomarkers identified include the estimated glomerular filtration rate, C-reactive protein, or plasma B-type natriuretic peptide, which reflect renal dysfunction, systemic inflammation, and cardiac haemodynamic burden, respectively [29,30,31]. The capacity of these biomarkers to quantify a patient’s disease state can improve utility in predicting AF recurrence following catheter ablation compared to their clinical counterparts. Variables of cardiac anatomy and function, determined using various cardiac imaging modalities, demonstrate significant promise in predicting the recurrence of AF after catheter ablation [27]. Acknowledging the multifaceted contributions of cardiac imaging to clinical practice and patient care [32], they will be discussed in detail later in this review. In brief, measures of cardiac structure, such as left atrial (LA) volume and diameter, both reflect and quantify the LA dilatation from the atrial remodelling seen in AF. In fact, increased LA volume and diameter is associated with increased risk of AF recurrence [27]. Similarly, measures such as the LA ejection fraction and LA strain help clinicians to ascertain an individual’s atrial function, which can indicate disease progression [27,33]. 

### 2.2. Scoring Systems for Predicting AF Recurrence

While previously mentioned measurements and variables provide some prognostic value, no single variable can accurately predict AF recurrence. Consequently, prognostic scoring systems and nomograms have been developed to provide a holistic and individualised risk assessment for patients suitable for catheter ablation (Table 1). The most common predictors used in these scoring systems were age, AF type, and LA parameters such as LA volume and LA diameter. A comparison of all the scoring components used in each scoring system is provided in Supplementary Table S2. Early recurrence of AF was included in scoring systems (BASE-AF_2_ and MB-LATER) that were designed for post-procedural prediction of AF recurrence [26,34]. BASE-AF_2_ and MB-LATER demonstrate high performance compared to other scoring systems (Table 1), achieving an area under the receiver operator characteristic curve (AUROC) of 0.94 and 0.782, respectively [35,36]. However, the use of early recurrence of AF as a scoring component may limit the use of these scoring systems in the preprocedural context where patient selection is being evaluated. 

Although scoring systems can be informative for the management of patients undergoing catheter ablation, they each possess their own strengths and weaknesses and require further validation, with systematic reviews unable to establish or suggest a single ‘universal score’ for use in clinical practice [34]. Hence, scoring systems are selected and used at the discretion of a clinician, and in conjunction with their personal clinical expertise and experience. In general, it is acknowledged that a key strength in the clinical utility of these scores comes from their ease of use, allowing for the standardisation of clinician decision-making [34]. Moreover, these scores are derived from predictors that have been validated and verified in clinical trials, allowing for them to be easy to understand for both patients and clinicians [26]. However, it is the same simplicity of these scoring systems that may be holding back the potential performance of these models, as they are often limited to a small number of scoring components and are unable to comprehensively personalise the risk estimation for the patient. Although the initial reported performance of a particular scoring system may be promising (Table 1), validation studies conducted in different population groups are highly variable but often marked by a drop-off in performance [26]. Despite this, prognostic scores demonstrate that tools developed using statistical methods that can consider a variety of predictors of AF recurrence perform better compared to the use of these predictors alone, due to the individualised prognostication that is performed [26].

## 3. Cardiac Imaging for Predicting AF Recurrence

Over the years, the use of cardiac imaging to assess the structure and function of the heart has become a fundamental element of routine clinical care in cardiovascular medicine [32,48]. It is understood that cardiac imaging can prove pivotal in predicting AF recurrence following catheter ablation. Accordingly, a wide range of cardiac imaging modalities is used in the care of patients with AF, including echocardiography, CCT, CMR imaging, positron emission tomography and fluoroscopy [32]. For many of these modalities, standard measurements such as LA volume or left ventricular ejection fraction play important roles in routine clinical practice. However, the unique capabilities of each modality may not be harnessed as these standard measurements can be determined by several different imaging modalities [14,32]. As this review aims to explore the state-of-the-art advancements in cardiac imaging, a focus will be placed on concepts and measurements that showcase the growing strengths of imaging technologies.

### 3.1. Echocardiography

Echocardiography is a key imaging modality used in the care of patients with AF. The three main techniques for echocardiographic imaging are transthoracic echocardiography (TTE), transoesophageal echocardiography (TOE) and intracardiac echocardiography. Intracardiac echocardiography remains a developing technology within the context of predicting AF recurrence after catheter ablation and is hence outside of the scope of this review. A key strength of echocardiography lies in its high temporal resolution, allowing for accurate measurement of Doppler velocities and cardiac function. Among echocardiographic techniques, TTE stands out for its non-invasiveness, speed, and ease of use. On the other hand, TOE provides improved visibility of posterior cardiac structures such as the left atrium [49]. Important considerations of echocardiography include the higher inter- and intra-observer variability compared to CMR, which can be attributed to operator-dependent variability in image quality [49]. Developing technologies such as 3-D echocardiography may mitigate this limitation but have yet to be used in studies predicting AF recurrence to the best of our knowledge. 

LA strain is an independent measure of LA function that takes advantage of the temporal resolution of TTE and TOE and can be acquired by using 2-D STE [49,50]. LA strain is defined as the relative percentage deformation of the LA during a single heart cycle. Consequently, LA strain allows for measurement of the heart’s diastolic function, and has demonstrated the ability to identify subclinical diastolic dysfunction [50]. Furthermore, a decreased LA strain predicts increased LA fibrosis, which is seen with increased atrial remodelling during AF, allowing for interpretation of a patient’s AF disease state [50]. Studies have delineated the association between LA global longitudinal strain (GLS) and AF recurrence after catheter ablation, such that LA GLS is decreased in patients with recurrent AF [51,52]. A meta-analysis conducted by Mouselimis et al. included 880 patients and determined that a pooled LA GLS cut-off value of 21.9% showed predictive power for AF recurrence. In their analysis, LA GLS outperformed classical echocardiographic parameters such as LA diameter and LA volume index [51]. 

**Figure 1:**
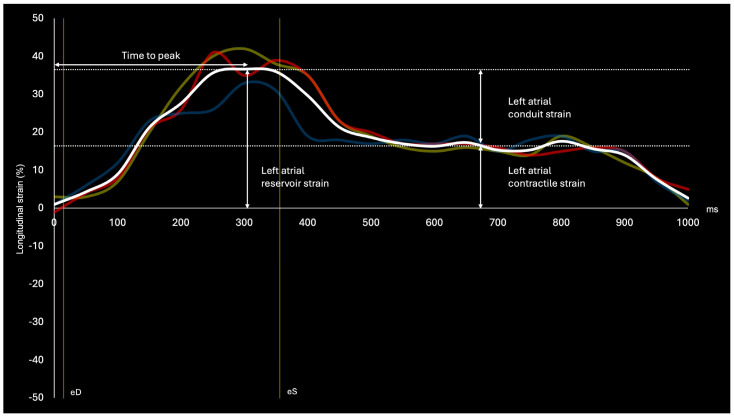
Annotated diagram of a left atrial (LA) strain curve during normal sinus rhythm. The strain curve highlights reservoir (atrial filling), conduit (ventricular filling), and contractile (atrial emptying) phases of the LA cardiac cycle. LA left wall strain (green), LA right wall strain (blue), LA roof strain (red), and average LA strain (white) are shown. eD: end diastole; eS: end systole.

LA strain can also be graphed as a curve, divided into three phases to represent the LA cardiac cycle, shown in Figure 1, with corresponding measurements of LA reservoir strain (atrial diastole), LA conduit strain (ventricular diastole), and LA contractile strain (atrial systole). Furthermore, additional measurements can be made from the strain curve, including time to peak and strain measurements separated by region (right wall, left wall, roof). Similarly, the derivative of LA strain, known as LA strain rate, has demonstrated merit in assessing LA function [53]. Each of these parameters may have further predictive value in the context of AF recurrence after catheter ablation but require further validation in systematic contexts.

### 3.2. Cardiac Computed Tomography and Cardiac Magnetic Resonance Imaging

In addition to echocardiography, CCT and CMR imaging are used extensively to study the anatomy of the heart prior to catheter ablation [14]. In contrast to echocardiography, CCT and CMR provide high spatial resolution, allowing for detailed and accurate image acquisition. In addition to TOE, these imaging modalities may also be used for the exclusion of LA thrombus, a key contraindication to catheter ablation. Also, CCT and CMR provide a comprehensive anatomical assessment of the PVs [32]. Importantly, atypical pulmonary vein anatomy may increase the likelihood of AF recurrence in patients undergoing cryoablation [32].

CCT can also be used for the analysis of atrial adipose tissue, which is a known marker of local inflammation [8,32]. As inflammation is closely related to the complex aetiology of AF, it has been hypothesised that epicardial tissue volume can be used to predict AF recurrence after catheter ablation. Teixeira et al. analysed 350 patients and found that an epicardial adipose tissue volume above 92 cm^3^ predisposed patients to a two-fold increase in risk of AF recurrence after catheter ablation [54]. These findings demonstrated independent predictive value compared to other clinical and imaging risk factors. As a single-centre study, further research is required for the development of a reference cut-off value for epicardial adipose tissue volume in predicting AF recurrence after catheter ablation. 

Furthermore, the high spatial resolution of CCT allows for the acquisition of radiomic features that quantify tissue heterogeneity from raw CCT images. Radiomics describes a feature extraction technique that allows for the identification of changes or relationships that were unidentifiable when considering the raw image alone [55]. Hence, radiomics analysis using CCT images may be useful in predicting AF recurrence, and in identifying complex features that differentiate patients at risk of AF recurrence after catheter ablation. Despite the promise and growth of radiomics research, further study is required to validate these measurements in a large multi-centre cohort of patients with AF [56,57]. 

CMR provides an alternative to CCT for imaging the anatomy of the LA and PVs. CMR can also provide additional quantitative analysis of LA fibrosis. Using late gadolinium enhancement and CMR, clinicians can measure the amount of fibrotic atrial tissue and quantify the level of LA fibrosis present. As atrial fibrosis is the predominant consequence of the atrial cardiomyopathy driven by AF, the ability to quantify a patient’s atrial fibrosis prior to catheter ablation may be useful in predicting AF recurrence [8,32]. This is consistent with meta-analysis conducted by Regmi et al., who delineated that the quantification and staging of atrial tissue fibrosis, as determined from CMR, is strongly associated with AF recurrence after catheter ablation [58,59]. However, Regmi et al. highlighted the significant variability in cut-off values for classifying fibrosis level between studies [59]. 

Regarding strain calculations with CCT and CMR, advances in feature tracking techniques and technologies have allowed for strain measurements to be conducted using CCT and CMR images. Considering the increased spatial resolution and decreased intra- and inter-observer variability of CCT and CMR, strain measurements derived from CCT and CMR may be ideal predictors of AF recurrence following catheter ablation [60,61]. Comparison studies have noted a strong correlation between LA strain derived from CMR and CCT feature tracking, and LA strain derived from 2-D STE. Despite this, the ease of use and superior temporal resolution of 2-D STE underscore its use as the gold standard for strain and functional studies [60,61]. 

## 4. Applications of Artificial Intelligence for Predicting AF Recurrence

Over time, AI has been used increasingly in all aspects of modern medicine, ranging from the production of clinical decision-making tools to automated interpretation of genetic sequencing [62,63]. Use of AI is particularly pertinent in cardiology, a field of medicine in which clinicians are exposed to, and work closely with, a diverse range of data modalities, ranging from various clinical, imaging, electrophysiological or ‘-omic’ data [62]. Increasing use of ’big data’ as health systems transition into fully digital formats has driven a necessity for AI in cardiology, and various advances have since been made in recent history [62]. With these data, AI can automate time-consuming tasks without fatiguing and identify complex relationships that would have been missed by the human eye. Overall, the implementation of AI into clinical practice does not aim to replace clinicians, but rather help clinicians keep up with an increasingly digital world [62,63,64,65].

Machine learning (ML) is a branch of AI where computational and statistical algorithms undergo continuous iteration and development involving the detection and analysis of patterns in structured input data. These algorithms, also known as models, are thus able to learn without any explicit programming, and are equipped to generate predictions for new unseen data when provided with the same input characteristics [64,66,67]. Deep learning (DL) is a subdomain of ML describing AI methods that draw inspiration from the neuronal network of the human brain. It involves the use of multiple statistical and computational layers, both explicit and hidden, to form an artificial neural network that is tailored to work with unstructured, high-dimensional data, such as image or natural language [62,66]. Consequently, DL aims to not only tackle ML tasks such as classification, regression, or clustering, but also more complex tasks such as natural language processing and computer vision.

### 4.1. AI Models to Predict AF Recurrence

**Figure 2:**
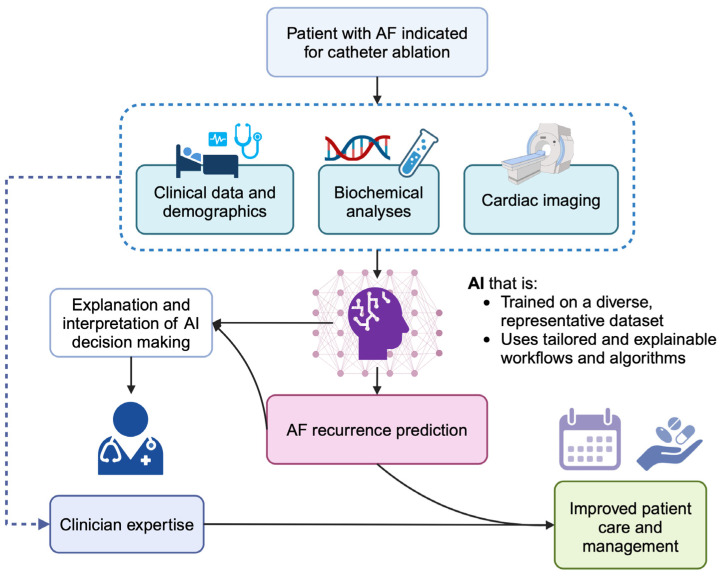
Hypothetical workflow demonstrating how artificial intelligence (AI) can be used to improve clinician decision-making and patient care for patients with atrial fibrillation (AF).

The ability of AI classification to integrate complementary data modalities and learn nuanced patterns equip ML models with the potential to accurately predict the recurrence of AF following catheter ablation. It is hypothesised that AI, once validated and used appropriately, will be able to integrate seamlessly into clinician workflows to streamline and optimise patient care (Figure 2). Moreover, the use of AI techniques has the potential to identify sub-populations suitable for first-line catheter ablation, which was previously unrevealed from standard statistical approaches [68]. 

The use of AI to predict AF recurrence has been investigated in several studies, although external validation of model efficacy remains a key barrier to widespread use [69]. In these studies, ML methods were used to create prediction scores and prognostic models for the recurrence of AF after catheter ablation [69]. In terms of prediction scores, researchers have employed unsupervised clustering models to determine the features with the highest predictive ability and integrated these features within a scoring system [70]. On the other hand, many studies have extended the use of ML, using supervised ML models to complete the classification task in an end-to-end manner, from feature selection to the final prediction of ‘no recurrence’ or ‘recurrence’. These approaches have been detailed comprehensively and use a range of data modalities and AI model frameworks (Table 2). In Table 2, the performance of these models has been reported using AUROC. The AUROC is a widely used metric used in ML studies to summarise an AI classification model’s performance [71]. Additional performance analysis using the sensitivity, specificity, recall, the F1 score, or Kaplan–Meier survival analysis is both helpful and important for providing a holistic understanding of an AI model’s behaviours [72,73]. By understanding an AI model’s behaviours, strengths and biases, clinicians are equipped to judge the clinical scenarios where the use of AI is most applicable. In addition to these metrics, the calculation of confidence intervals and other uncertainty analyses are beneficial to guide clinician’s judgement of how trustworthy the AI model’s decision may be prior to validation studies [73].

Previous models utilising cardiac imaging (TTE/TOE, CMR and CCT) have achieved promising performance using a combination of ML techniques [33,81,88]. Many studies conducted basic statistical analysis involving univariate and multivariate logistic regressions, all reporting an improved performance in predicting AF recurrence post-catheter ablation when using an ML technique over their traditional statistical counterparts [56,70,76,78,79,87,91]. Tang et al. and Lee et al. compared their ML models directly against the CHA_2_DS_2_-VASc and APPLE scores, scores informed by studies using traditional statistical tests, and demonstrated incrementally improved performance [82,85]. Miao et al. utilised a Least Absolute Shrinkage and Selection Operator model trained using standard echocardiographic measurements from a cohort of 403 patients to achieve an AUROC of 0.878 [33]. Roney et al. trained a random forest model on CMR scans and heart simulations derived from these scans to achieve an AUROC of 0.85 from a comparatively small cohort size of 100 [81]. Finally, a recent study by Razeghi et al. utilised the unique strengths of DL to develop a multimodal fusion framework model that combines clinical, CCT scans and fractal feature data from 321 patients to predict AF recurrence after catheter ablation with an AUROC of 0.821 [88]. These studies demonstrate the benefits of integrating cardiac imaging into AI models, providing superior performance to models using clinical data alone. However, all three of these studies were single centre studies, raising questions around external validity and generalisability [33,81,88]. Although each of these studies employed advanced sampling and validation techniques such as synthetic minority oversampling technique and k-fold cross validation to mitigate the effect of their smaller sample sizes and to reduce overfitting of their ML models, the performance of ML models remains inherently data driven. Therefore, training on smaller datasets conducted in single centres can produce biased models that demonstrate poor performance when applied to external contexts and new patient populations [95]. While these performance metrics are not necessarily misleading, similar performance is unlikely to be achieved in broader populations.

To the best of our knowledge, the study conducted by Saglietto et al. is the largest study to date aiming to predict the recurrence of AF after catheter ablation using an ML approach, capturing 3128 patients from 104 centres. Utilising clinical and demographic data variables from the ESC EORP AFA-LT registry to train and test a random forest model, their model achieves a calibrated AUROC of 0.721. This suggests an attenuation of AI model performance when applied to general use; however, the prediction model still outperforms their scoring system counterparts. Notably, their model was then developed into a clinician-friendly web calculator, AFA-Recur, allowing for widespread use and validation [83]. Integration of cardiac imaging modalities could be an area of research that may boost model performances. 

While the number of studies investigating the use of radiomic features or CMR simulations is growing, only a single study has considered LA strain in an ML prediction model for AF recurrence following catheter ablation [76]. It is noted however, that other studies have considered the utility of 2-D STE to measure LA strain for use in ML models for risk stratification of patients with heart failure [96]. With regard to AF recurrence, Hwang et al. studied 606 patients, developing a convolutional neural network model that interprets M-mode strain images in conjunction with standard echocardiographic measurements, achieving a test performance of 0.796 [76]. Hwang et al. highlighted the superior predictive value of LA strain over standard echocardiographic parameters alone. 

### 4.2. Limitations of AI in Medicine

Ethical considerations remain a key barrier to the widespread adoption of AI in clinical practice. Two primary ethical concerns are the ‘black-box problem’ and potential bias in AI models. The ‘black-box problem’ refers to the decreasing capacity for humans to understand the decision-making process of AI systems as they become increasingly advanced [66,97]. Hence, attempts to improve the explainability of AI models has been a focus of research, particularly in prediction models, ensuring that they act as clinical decision-making aids, rather than as clinician surrogates. An emerging concept in AI is ‘interpretable’ AI, that is, AI that is specifically designed to transparently output its decision-making schema concurrently with its intended output, allowing for direct interpretation of AI decision-making without the need for post hoc explainability analysis using techniques such as saliency maps or Shapley Additive Explanations [98].

Another ethical consideration for AI is the potential for bias. As each model is trained on datasets to make predictions or perform different tasks, if this dataset does not appropriately represent the intended user population, then the accuracy of any resulting predictions or assertions is severely compromised. Hence, it can be appreciated that AI models remain largely hypothesis-generating for the time being, with the potential to drive further research as technologies improve [66,97]. The potential for model bias is especially important when considering the use of ML models in different patient populations. Already seen with non-AI-based cardiovascular risk prediction models, such as the Framingham Risk Score, development of these prediction models without involvement or analysis of diverse population cohorts can perpetuate inequalities in medical care [97]. The same is to be said with AI models, where underrepresentation of minority groups during model training data can lead to poor estimation of risk, and adverse clinical outcomes [62,97]. The use of large, diverse, multi-centre datasets in ML model training is henceforth essential to prevent the exacerbation of current inequalities in medicine [64]. When considering the development or use of larger datasets, concerns arise surrounding informed consent, the use of identifiable patient data for training AI models, and potential data leakages. Therefore, the large data requirements of AI and consequent minimisation of model bias needs to be balanced with patient privacy [97]. Data encryption and deidentification, federated learning and strong data custodianship all describe strategies with different merits and shortcomings, all aiming to balance privacy concerns with the need for big data in AI model training [97,98]. In the same way that ML is helping clinicians keep up with big data, big data must also keep up with and facilitate the development of ML models.

## 5. Future Directions

Despite extensive research on the prediction of AF recurrence following catheter ablation, there are still gaps that sustain AF recurrence at the forefront of research focus. Several questions remain, particularly in the integration of novel predictors from cardiac imaging with developing AI technologies. Firstly, while LA GLS and its relationship with AF recurrence has been well documented in previous studies [51,52], other aspects of LA strain such as regional LA strain and LA strain rate remain underutilised. In addition, the strain curve itself may hold untapped potential given the strengths of AI to interpret time series data, as demonstrated by Ntalianis et al. [99]. The ability of ML to integrate time-series data provides an avenue for the incorporation of complete strain curves into ML models, potentially providing increased prognostic value over peak strain values alone. Other metrics derived from the strain curve, such as time to peak, require further research but have also demonstrated potential in preliminary studies [100]. Similarly, there have been a growing number of studies investigating the use of radiomics features from CCT imaging or simulations from CMR imaging [56,74]. These approaches capitalise on the unique advantages of AI to consider high-dimensional data, and further work with these approaches, especially with validation in larger cohorts, is desirable. 

**Figure 3:**
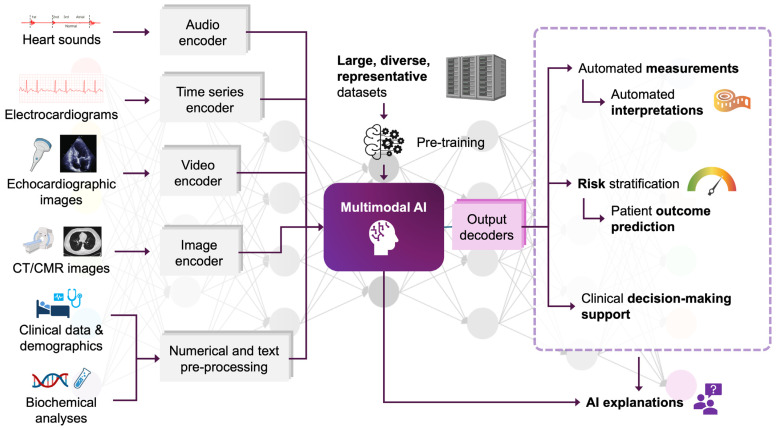
A multimodal AI framework for the personalised care of patients with atrial fibrillation. CCT, cardiac computed tomography; CMR, cardiac magnetic resonance; AI, artificial intelligence.

Moreover, few AI studies address the lack of explainability, particularly in the context of DL and radiomics. Future research should focus on AI explainability, as increased explainability can mitigate the ‘black box effect’ that is present in AI models and allow for greater autonomy for clinicians when using the model in clinical practice [62,97,101,102]. A growing body of work provides a foundation for continued development of ‘explainable AI’, with Shapley Additive Explanation analysis and other interpretative techniques already being used [86,90]. This is especially relevant given that AI and ML models are becoming increasingly ‘multimodal’, which describes the capacity of AI models to consider and analyse multiple data input modalities simultaneously [103]. Multimodal AI models are promising in providing improved performance and workflow integration by leveraging multiple forms of data, rather than focusing on a single imaging or data modality alone [67]. This capability extends to data modalities outside the scope of this review, including heart sounds and electrocardiograms [62,67]. Figure 3 accordingly demonstrates a multimodal framework that may be developed in the future to leverage AI to its fullest. In the same manner that research transitioned from single predictors to prediction scores many years ago, the research landscape is likely to transition once more from single modality AI models to multimodal AI models to provide evidence-based, personalised patient care. 

Finally, the generalisability and external validity of AI models represent a key factor when considering the translational impact of AI in medicine. While a trend towards multi-centre and prospective trials investigating AI-based prediction tools has been observed [92,93,94], large-scale clinical trials that implement these AI tools in periprocedural care patients with AF undergoing catheter ablation will be crucial to externally validate the performance and AF recurrence risk prediction reported in previous ML studies. Furthermore, clinical trials can help researchers overcome barriers to implementing AI into clinical practice by highlighting the tangible impacts of AI in healthcare systems, driving potential changes to current infrastructure and clinical workflows. Therefore, while the recent rise of AI in medical research is certainly promising, clinical trials investigating outcomes after implementation to clinical practice are essential in ensuring that patients and clinicians can reap the rewards of emerging technologies in AI and multimodality cardiac imaging [104,105]. 

## 6. Conclusions

The recurrence of AF remains a significant challenge for patients undergoing catheter ablation. Prediction models driven by AI and cardiac imaging can empower clinician decision-making to allow for improved patient selection and management. The generalisability and explainability of AI models remain key limitations to widespread use in clinical practice. Ongoing research into AF recurrence after catheter ablation and its prediction can provide key insights that may help researchers improve the accuracy of prediction models by employing cutting-edge technologies, potentially leading to improved patient outcomes. 

## Figures and Tables

**Table 1:** Summary of publications detailing the use of scoring systems to predict the recurrence of atrial fibrillation (AF) following catheter ablation.

Scoring System (Year) [Reference]	Scoring Components	AUROC
ALARMEc (2013) [37]	**A**F type (non-paroxysmal) Normalised **LA** area **R**enal insufficiency **Me**tabolic syndrome **C**ardiomyopathy	0.66
BASE-AF_2_ (2013) [36]	**B**ody mass index **A**trial dilatation **S**moking **E**arly recurrence of AF **AF** duration **AF** type (non-paroxysmal)	0.94
CHADS_2_ (2014) [29]	**C**ongestive heart failure **H**ypertension **A**ge (>75 years) **D**iabetes mellitus Prior **s**troke/TIA/TE	N/A
CHA_2_DS_2_-VASc (2014) [29]	**C**ongestive heart failure **H**ypertension **A**ge (>75 years) **D**iabetes mellitus Prior **s**troke/TIA/TE **V**ascular disease **A**ge (>65 years) **S**ex **c**ategory (Female)	0.55
APPLE (2015) [38]	**A**ge (>65 years) **P**ersistent AF Im**p**aired eGFR **L**A diameter LV**E**F	0.63
CAAP-AF (2016) [39]	**C**oronary artery disease L**A** diameter **A**ge **P**ersistent or long-standing persistent AF Number of **A**ADs failed **F**emale sex	0.65
MB-LATER (2017) [35]	**M**ale gender **B**undle branch block **LA** diameter AF **t**ype **E**arly **r**ecurrence of AF	0.78
ATLAS (2018) [40]	**A**ge (>60 years) AF **t**ype (non-paroxysmal) **LA** volume indexed **S**ex category (Female) **S**moking	N/A
LAGO * (2018) [41]	AF type (non-paroxysmal) Structural heart disease CHA_2_DS_2_-VASc LA diameter LA sphericity	0.69
HEAL-AF (2020) [42]	**H**eart failure **E**lderly (>75 years) **A**symptomatic AF **L**ong-standing persistent AF **A**trial dilation **F**emale sex	0.72
FLAME (2021) [43]	**F**emale **L**ong-lasting persistent AF **A**trial (left) diameter **M**itral regurgitation **E**xtreme comorbidities	0.69
HASBLP (2022) [44]	**H**istory of AF **A**ge **S**noring **B**ody mass index Anteroposterior **L**A diameter **P**ersistent AF	0.78
C2HEST (2023) [45]	**C**ongestive heart failure **C**hronic obstructive pulmonary disease **H**ypertension **E**lderly (age ≥ 75) **S**ystolic heart failure **T**hyroid disease	0.88
VAT-DHF (2023) [46]	**V**olume **A**F **t**ype **D**iabetes **H**eight **F** waves	0.87
HeLPS-Cryo (2024) [47]	**H**eart failure **L**eft atrial diameter > 40mm **P**ersistent AF **S**troke	0.89

* LAGO: left atrial geometry and outcome. Bold font is used to denote characters used for the naming of each scoring system. AAD, antiarrhythmic drug; AF, atrial fibrillation; AUROC, area under receiver operator characteristic curve; BMI, body mass index; eGFR, estimated glomerular filtration rate; LA, left atrial; LVEF, left ventricular ejection fraction; TE, thromboembolism; TIA, transient ischaemic attack.

**Table 2:** Summary of publications involving the use of artificial intelligence to predict the recurrence of atrial fibrillation after catheter ablation.

Publication (Year) [Reference]	Sample Size	AI Model(s) Used	Data Source(s) Used	Key Model Features	AUROC
Shade et al. (2020) [74]	*n* = 32 Single centre	QDA	CMR	Simulated features ^a^ (reentry and pacing locations)	0.82
Kim et al. (2020) [75]	*n* = 527 Single centre	CNN	Clinical	3D LA reconstructions, LA volume	0.61
Hwang et al. (2020) [76]	*n* = 606 Single centre	LR, CNN	Clinical, TTE	LA diameter, LA ejection fraction, LA strain	0.80
Firouznia et al. (2021) [57]	*n* = 203 Single centre	RF	Clinical, CCT	Radiomic features ^b^ of LA and PVs, AF type	0.81
Atta-Fosu et al. (2021) [77]	*n* = 68 Single centre	XGB and CNN	Clinical, CCT	LVEF, age	0.78
Lee et al. (2021) [78]	*n* = 2881 Multi-centre (primarily single centre)	CNN	Clinical, CCT	LA wall stress, AF type	0.73
Miao et al. (2021) [33]	*n* = 403 Single centre	LASSO, LR	Clinical, TTE	AF duration, LA volume indexed, LA expansion index, LA emptying percentage index	0.88
Labarbera et al. (2021) [79]	*n* = 66 Single centre	LDA, QDA, SVM, RF	Clinical, CCT	Age, hypertension, radiomic features of LA and PVs	0.70
Zhou et al. (2022) [80]	*n* = 310 Single centre	CNN	Clinical, CCT, TTE	NT-proBNP, AF type, LA appendage volume, LA volume	0.76
Roney et al. (2022) [81]	*n* = 100 Single centre	kNN, SVM, RF, LR	Clinical, CMR	Simulation features ^a^, visual fibrosis score of PVs	0.85
Yang et al. (2022) [56]	*n* = 314 Single centre	RF, LR	Clinical, CCT	Radiomic features ^b^ of LA and LA epicardial adipose tissue, LA epicardial adipose tissue volume	0.85
Tang et al. (2022) [82]	*n* = 156 Single centre	CatBoost, CNN, MMFF	Clinical, ECG, electrogram	LVEF, BMI, LA surface area, LA volume	0.86
Saglietto et al. (2022) [83]	*n* = 3128 Multi-centre	RF, DT, AdaBoost, kNN	Clinical	Left ventricular end diastolic volume, eGFR, BMI, Age, LA diameter	0.72
Warminski et al. (2022) [84]	*n* = 250 Single centre	CNN	Clinical, ECG	ECG analysis, LA volume, LVEF	0.76
Lee et al. (2022) [85]	*n* = 177 Single centre	LR, XGB, SVM, MLP	Clinical, TTE, biochemical	AF duration, Left ventricular mass indexed, eGFR	0.77
Ma et al. (2023) [86]	*n* = 471 Single centre	RF	Clinical	ERAF, hypertension, AF duration, LA diameter, age	0.67
Jiang et al. (2023) [87]	*n* = 1618 Single centre	CNN	Clinical, ECG	LA enlargement, ERAF, ECG analysis	0.84
Razeghi et al. (2023) [88]	*n* = 321 Single centre	LR, SVM, RF, CNN	Clinical, CCT	Radiomic features ^b^ of LA and PVs, history of prior ablation, hypertension	0.82
Brahier et al. (2023) [89]	*n* = 653 Single centre	RSF, MVST	Clinical, CCT	LA volume indexed, ERAF	N/A
Horde et al. (2023) [70]	*n* = 476 Single centre	LR	Clinical	Atrial flutter, renal disease, LVEF, valvular heart disease	N/A
Budzianowski et al. (2023) [90]	*n* = 201 Single centre	DT, LR, RF, XGB, SVM	Clinical, biochemical	ERAF, TSH	0.75
Sun et al. (2023) [91]	*n* = 349 Single centre	XGB, LR, SVM, RF	Clinical, TOE	LA appendage ejection fraction, NT-proBNP, LA appendage global peak longitudinal strain	0.87
Li et al. (2024) [92]	*n* = 509 Multi-centre	RF, kMC, DT	Clinical, CCT	Morphological grouping from CCT, age, BMI, AF type	0.79
Peng et al. (2024) [93]	*n* = 306 Multi-centre	AutoGluon-Tabular	Clinical, procedural	AF type, ablation duration, number of ablation lesions	0.78
Liu et al. (2024) [94]	*n* = 638 Multi-centre	LR, SVM, CatBoost	Clinical, CCT	I-Score combined variables ^c^	0.76

^a^ Studies by Shade et al. and Roney et al. used CMR images to simulate the LA and PVs during initiation of AF [74,81]. ^b^ Studies by Firouznia et al., Labarbera et al., Yang et al., and Razeghi et al. used software to extract shape and texture-based fractal features from CCT images [56,57,79,88]. ^c^ Liu et al. used the I-score algorithm to combine and correlate binary variables [94]. AdaBoost, adaptive boosting; AF, atrial fibrillation; AUROC, area under the receiver operator characteristic curve; BMI, body mass index; CatBoost, categorical boosting; CCT, cardiac computed tomography; CMR, cardiac magnetic resonance; CNN, convolutional neural network; DT, decision tree; ECG, electrocardiogram; eGFR, estimated glomerular filtration rate: ERAF, early recurrence of atrial fibrillation; kMC, k-means clustering; kNN, k-nearest neighbours; LA, left atrial; LASSO, least absolute shrinkage and selection operator; LDA, linear discriminant analysis; LR, logistic regression; LVEF, left ventricular ejection fraction; MLP, multilayer perceptron; MMFF, multimodal fusion framework; MVST, multivariable survival tree; NT-proBNP, N-terminal pro-brain natriuretic peptide; PV, pulmonary vein; QDA, quadratic discriminant analysis; RF, random forest; RSF, random survival forest; SVM, support vector machine; TOE, transoesophageal echocardiography; TSH, thyroid stimulating hormone; TTE, transthoracic echocardiography; XGB, XGBoost.

## Supplementary Materials

The following supporting information can be downloaded at: https://www.mdpi.com/article/10.3390/jcdd11090291/s1, Supplementary Table S1. Comparison table of recent AF guidelines on the indications for catheter ablation. Supplementary Table S2. Comparison of scoring components used in scoring systems to predict the recurrence of atrial fibrillation (AF) following catheter ablation.
