# Supplementary material for: Beyond Clinical Factors: Harnessing Artificial Intelligence and Multimodal Cardiac Imaging to Predict Atrial Fibrillation Recurrence Post-Catheter Ablation
Source: J Cardiovasc Dev Dis. 2024 Sep 19;11(9):291. doi: 10.3390/jcdd11090291 (PMC11432286; doi:10.3390/jcdd11090291)
Supplement: Supplementary file 1 [file jcdd-11-00291-s001.zip › jcdd-3185816-supplementary.pdf]

Supplemental Material

Supplementary Table S1. Comparison table of recent atrial fibrillation (AF) guidelines on the indications for catheter ablation.

	**AHA** [7]	**EHRA** [8]	**CSANZ** [9]
**1**	**Patients with symptomatic AF, in whom AADs are ineffective/contraindicated/not tolerated/not preferred, but rhythm control is desired**<br><br>Select patients* with symptomatic paroxysmal AF where rhythm control is desired (first line)	**Patients with symptomatic AF, resistant or intolerant to >1 AAD**<br><br>Patients with symptomatic recurrent paroxysmal AF (first line)<br><br>Patients with AF and left ventricular systolic dysfunction, suspected to be related to arrhythmia-mediated cardiomyopathy	**Symptomatic paroxysmal or persistent AF refractory to AADs**<br><br>Select patients with AF and HFrEF where AF is thought to be the primary cause of ventricular dysfunction (irrespective of presence of symptoms)
**2A**	Patients with symptomatic paroxysmal or persistent AF where rhythm control is desired (first line)	Patients with AF and HFrEF<br><br>Patients with AF and symptomatic bradycardia or prolonged sinus pauses	Patients with symptomatic paroxysmal or persistent AF (first line)<br><br>Patients with concurrent AF and HFrEF
**2B**	**Select patients* with asymptomatic or minimally symptomatic AF**	**Select asymptomatic patients with recurrent AF following informed risk-benefit discussion**<br><br>Patients with symptomatic persistent AF	**Asymptomatic patients following informed risk-benefit discussion**<br><br>Symptomatic long-standing persistent AF refractory to AADs<br><br>Symptomatic long-standing persistent AF prior to AAD

* Patients that are generally younger with fewer comorbidities, and moderate to high burden of AF or persistent AF

(1) "TO DO"; (2A) "May be appropriate TO DO"; (2B) "Area of uncertainty". Areas of consensus across all three guideline documents are denoted in bold. AAD, antiarrhythmic drug; AHA, American Heart Association; CSANZ, Cardiac Society of Australia and New Zealand; EHRA, European Heart Rhythm Association; HFrEF, heart failure with reduced ejection fraction

Supplementary Table S2. Comparison of scoring components used in scoring systems to predict the recurrence of atrial fibrillation (AF) following catheter ablation.

Parameter	**ALARMEC** [37]	**BASE-AF$_2$** [36]	**CHADS$_2$** [29]	**CHA$_2$DS$_2$-VASc** [29]	**APPLE** [38]	**CAAP-AF** [39]	**MB-LATER** [35]	**ATLAS** [40]	**LAGO*** [41]	**HEAL-AF** [42]	**FLAME** [43]	**HAS-BLP** [44]	**C2HEST** [45]	**VAT-DHF** [46]	**HeLPs-Cryo** [47]	*Total*
AF type	✓	✓			✓	✓	✓	✓	✓	✓	✓	✓		✓	✓	**12**
Age			✓	✓	✓	✓		✓	✓	✓		✓	✓			**9**
LA diameter		✓			✓	✓	✓		✓	✓	✓	✓			✓	**9**
Female sex				✓		✓		✓	✓	✓	✓					**6**
Heart failure			✓	✓					✓	✓			✓		✓	**6**
DM/metabolic syndrome	✓		✓	✓					✓					✓		**5**
Hypertension			✓	✓					✓				✓			**4**
Stroke			✓	✓					✓						✓	**4**
Structural heart disease	✓			✓					✓		✓					**4**
AF duration		✓										✓				**2**
Body mass index		✓										✓				**2**
Early recurrence of AF		✓					✓									**2**
LA volume								✓						✓		**2**
Renal insufficiency/disease	✓				✓											**2**
Smoking		✓						✓								**2**

* LAGO includes age, female sex, heart failure, DM/metabolic syndrome, hypertension, stroke and structural heart disease due to having CHADS-VASc score as one of their scoring components.

Asymptomatic AF, bundle branch block, chronic obstructive pulmonary disease, coronary artery disease, F waves, height, LA sphericity, LVEF, male sex, mitral regurgitation, LA area, number of AADs failed, snoring, thyroid disorders were each only used in one scoring system. LA, left atrial; DM diabetes mellitus.
